# The efficacy of ilizarov method for management of long tibial bone and soft tissue defect

**DOI:** 10.1016/j.amsu.2021.102645

**Published:** 2021-07-31

**Authors:** Faisal Miraj, Ahmad Nugroho, Ivan Mucharry Dalitan, Melitta Setyarani

**Affiliations:** a Pediatrics Orthopaedics and Limb Reconstruction Division, Department of Orthopaedics and Traumatology, Fatmawati General Hospital, Jakarta, Indonesia; b Resident of Orthopaedics and Traumatology Department, Dr. Cipto Mangunkusumo Hospital, Jakarta, Indonesia/ Faculty of Medicine, Universitas Indonesia, Jakarta, Indonesia

**Keywords:** Non-union fracture of tibia, Long tibial bone defect, Soft tissue defect, Illizarov method

## Abstract

**Introduction:**

Patients with open fracture Gustillo-Anderson grade 3 had undergone several surgical procedures, but still ended up with expose long dead bone or infected. Illizarov method was used to address long bone and soft tissue defect after re-debridement with radical resection of long dead bone or infected segment.

**Methods:**

We included 14 patients (mean age: 30.86 ± 11.49) with non-union tibial fracture with long dead and infected bone segment who had undergone several debridement, bone grafting or spacer and soft tissue closure procedure due to open fracture of tibia grade 3. The subjects underwent re-debridement with radical resection of dead or infected bone segment followed by Illizarov method to perform bone transport procedure for bone defect filling and simultaneously restore severe soft tissue loss and bone lengthening procedure.

**Results:**

All subjects had achieved satisfactory results with mean docking period of bone transport 3.78 ± 0.54 months, union time at the docking side 7 (5.5–9) months. Soft tissue was covered and no recurrence of infection. Three subjects had Leg Length Discrepancy (LLD) of 1 cm, whereas the remaining had zero discrepancy. No significant pain was observed at final follow-up and 4 patients had ankle joint stiffness.

**Conclusion:**

The Illizarov method can effectively address long bone and soft tissue defects by distraction osteogenesis through bone transport procedure that filling the defect gradually without bone graft and simultaneously enhancing soft tissue closure without tertiary soft tissue procedure subsequently followed with bone lengthening procedure to correct the limb length discrepancy.

## Highlights

• Open fracture Gustilo Anderson type 3 often results in devastating problems.
• Several surgical procedures still ended as a non-union fracture with infected or dead bone segment.
• Long bone and soft tissue defect occurred following re-debridement and radical resection.
• Ilizarov method can effectively fill the long bone defect by bone transport procedure without using bone graft and simultaneously enhanced soft tissue closure.

## 1 Introduction

Fracture of the tibia is one of the most common in long bone fracture with the incidence of open fracture is 25 % [1]. Tibia is subcutaneous and easily loses its soft tissue cover in trauma. As such, open fractures frequently occur. Managing tibial open fracture requires advanced planning to reconstruct the bone with sufficient tissue coverage. Thus, the rate of complications associated with open tibial fractures is high; infection, non-union and limb loss are the major causes of morbidity [[1], [2], [3]]. Defect can be produced after massive debridement in the management of infected open fracture in the case of infected. Major soft-tissue defect of the muscles, tendons, and joints restricts the functional outcome of the leg. Treatment progression for the defect should be evaluated not only the defect closure physically but also the functional outcome [4]

To eliminate infection, it is critical to resect all necrotic bone and infected segments [5]. After we achieved control of infection, our goals are to achieve bony union, correct deformities, equalize limb length, and fill defects and bone gaps. Union needs three basic requirements: stable fixation, biological stimulation, and restored function. Stable fixation can be achieved by any external or internal fixation devices. Iliac crest bone grafting is the gold standard and provides osteoconductive, osteoinductive, and osteogenic material to give biological stimulus [6,7]. Currently the development of stem cells in fracture healing shows promises, but the treatment brcan't be accessed easily [8].

One of the most common complications in open tibial fracture is infected non-union of the tibia. Such condition poses many challenges to both treating surgeon and the patient, as it may lead to recalcitrant infection, complex deformities, the sclerotic bone ends, large bone gaps, shortening, and joint stiffness. They are easy to diagnose and difficult to treat [6]. Tibial non-union has been treated with a variety of surgical methods including plate osteosynthesis with bone graft, intramedullary nailing, and external ﬁxation. Although the use of internal ﬁxation is effective in the treatment of some cases of tibial non-union, these techniques have their limitations [9]. The incidence of infected non-union of open fracture is increasing, and more than 50% of cases occur after internal fixation [10].

In contrast to other methods, the Illizarov technique is reported to address not only the bone defect but also the associated problems of shortening, deformity, soft-tissue loss, and joint contractures proving it as the best method as a limb salvage reconstruction method following a devastating trauma to the tibia. The dynamic frame enables gradual lengthening, deformity correction, and nonunion or delayed union compression using minimally invasive procedure [11,12]. The Illizarov method can also be used as a single staged procedure [13,14]. The outcome can be improved if early osteosynthesis attempted with the Illizarov method rather than when it was used after following of failed internal fixation [15].

The Illizarov method is very important invention in the field of orthopaedic to treat various bone disease and shortening. It can permit tension stress that provides adequate blood supply, stimulates tissue biosynthetic activity and callus formation, corrects gradual length, and early limb function and load.

Technique in Illizarov application is not aggressive, little blood loss, and soft tissue surrounding preservation. Post-operative intensive care is not required for this technique. The several important factors to produce excellent outcome of Illizarov are rigid in both circular and pillar connection of external fixation, fragment position management, bone transport, and bone regeneration evaluation. It can produce expected correction during the course of treatment for consideration to permit limb weight-bearing and maintain joint motion [[16], [17], [18]]. The other advantage of Illizarov treatment is that the patient can move the treated limb actively to increase physiological function and stimulate bone healing. Thus, it can minimize the risk of muscle atrophy and disuse osteoporosis occurrence [19].

The Illizarov fixator has revolutionized the treatment of infected tibial nonunions [6]. A meta-analysis by Peng Yin et al. concludes that the Illizarov method is a good choice for the treatment of infected non-union of the tibia [20]. In a meta-analysis of lower limb segmental defects treated by bone transport, the overall union rate was 95 % (range, 60–100 %) [21,22]. The studies further strengthen the choice of Illizarov Method in treating non-union tibia in our developing country, due to its reusable versatile design is very cost-effective. Illizarov ring fixator remains an excellent treatment modality for tibial nonunion with a defect, regarding bone union, deformity correction, infection eradication, limb-length achievement, and limb function [[23], [24], [25], [26], [27]].

The purpose of our study was to assess the efficacy of the Illizarov method in addressing both bone and soft tissue defect in patients with a history of open fracture or dead and infected bone segment and severe soft tissue injury who had undergone multiple surgeries.

## 2 Material and methods

This was a retrospective case series study of patients diagnosed with non-union tibial fracture due to open fracture tibia grade 3 who had undergone multiple surgeries between January 2015 to September 2019. The inclusion criterion was non-union tibial fracture with exposed long dead bone or infected bone segment with minimal defect 4 cm and poor soft tissue coverage, that had been confirmed clinically and by x-ray. Patients with non-trauma cases (e.g. pathological fracture), any additional pathology and loss of follow up were excluded from the study.

During clinical assessment, we abstracted the following patients’ information: age, sex, initial diagnosis, and what operations they had undergone before referred to our centre, we also review their previous surgical reports. Non-viable bone and soft tissue were radically resected; this resulted in long bone and soft tissue defects. Long bone defect also produced potential empty space that allowed primary closure of the soft tissue with subcutaneous undermining or rotation flap if needed. However, in some circumstances where primary closure could not be performed or dehiscence, secondary healing was enhanced by open treatment. Subsequently, Illizarov frame was utilized for bone transport procedure that started one week after the installation of the frame with the speed 1 mm/day divided into four times. Near the end of the transport the skin become infolded into docking site, we then surgically intervened to facilitate the docking by skin fold soft tissue reconstruction and did compression at the docking site. No bone grafting performed at the beginning nor the end of the procedure. The patients were encouraged to undergo partial weight-bearing 4 weeks after operation. Bone lengthening then performed to correct the limb length discrepancy. After the length was achieved, patients underwent Full Weight-bearing 2–3 months after Illizarov frame removal. Post-operative evaluation was performed using Foot and Ankle Disability Index (FADI) score and International Knee Documentation Committee (IKDC) score [28,29]. This case series has been reported in line with the PROCESS Guideline [30].

## 3 Results

14 patients were included in our study, 12 males and 2 females, age ranging from 12 to 47 years (mean age of 30.86 years, see Table 1). All of the patients had 6.5 cm–15 cm bone defect (mean 14.07 ± 4.37 cm), thus, were indicated for bone transport procedure. Most of the patients had undergone several operations before referred to our centre. All patients had bone exposed, six of the patients also had undergone failed regional flaps due to the severity of the soft tissue injury or infection.

**Table 1:** Demographic characteristics of the subjects.

Name	Age	Sex	Mechanism Injury	Previous Operation	Bone exposed	Bone Defect	Previous LLD (cm)	Docking Period (Months)	Union period (months)
ARP	21	M	Motor vehicle accident	Debridement, external fixation	2 × 3cm	10	1	3	6
AS	13	M	Motor vehicle accident	Debridement, external fixation, skin graft	4 × 5cm	19	2	6.5	10
EY	28	M	Motor vehicle accident	Debridement, external fixation, bone graft	3 × 4cm	12	1	4.5	8
MA	12	M	Motor vehicle accident	Debridement, external fixation	3 × 5cm	16	1	6	10
ME	47	M	Motor vehicle accident	Debridement, external fixation	2 × 4cm	23	2	9	13
MY	43	F	Motor vehicle accident	Debridement, external fixation	3 × 5cm	22	1	7.5	11
M	29	M	Motor vehicle accident	Debridement, external fixation, bone spacer	3 × 4cm	15	3	5	9
BR	33	M	Motor vehicle accident	Debridement, external fixation, Soleus Flap	4 × 5cm	13	1	4.5	8
MA	19	M	Motor vehicle accident	Debridement, external fixation, Gastroc flap	4 × 5cm	12	4	4	7.5
MS	46	M	Motor vehicle accident	Debridement, external fixation, bone spacer, Gastroc flap	3 × 4cm	12	1	4	8
GB	34	M	Motor vehicle accident	Debridement, external fixation, bone graft	2 × 4cm	10	2	4	7
F	40	M	Motor vehicle accident	Debridement, external fixation	3 × 5cm	12	2	4	8
NA	12	F	Motor vehicle accident	Debridement, external fixation	2 × 5cm	10	3	3.5	7
AG	35	M	Motor vehicle accident	Debridement external fixation	3 × 4cm	11	1	4	8
	30.86 ± 11.49^1^					14.07 ± 4.37	1.78 ± 0.97	4.96 ± 1.69	8.60 ± 1.84

Data are presented as ^1^mean ± standard deviation if the distribution is normal and presented as ^2^median (minimum to maximum) if the distribution is not normal. Normality test was performed using Kolmogorov-Smirnov test.

**Fig. 1:**
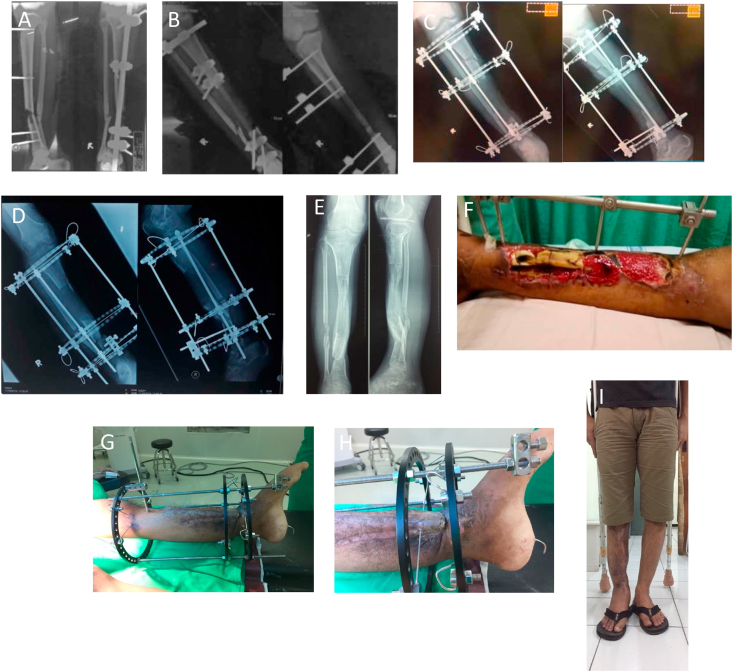
Case Illustration of a patient with severe soft tissue defect (bone exposed and non vital bone). (a) Initial radiology (b) post debdridement radiology with large bone defect (c) Illizarov application (d) Post bone transport and docking downward with skin fold soft tissue reconstruction followed by compression (e) Post Illizarov removal radiograph (f) initial clinical picture (g) healed soft tissue (h) infolded skin requiring soft tissue reconstruction (i) Post Illizarov removal clinical picture (0 cm LLD).

**Fig. 2:**
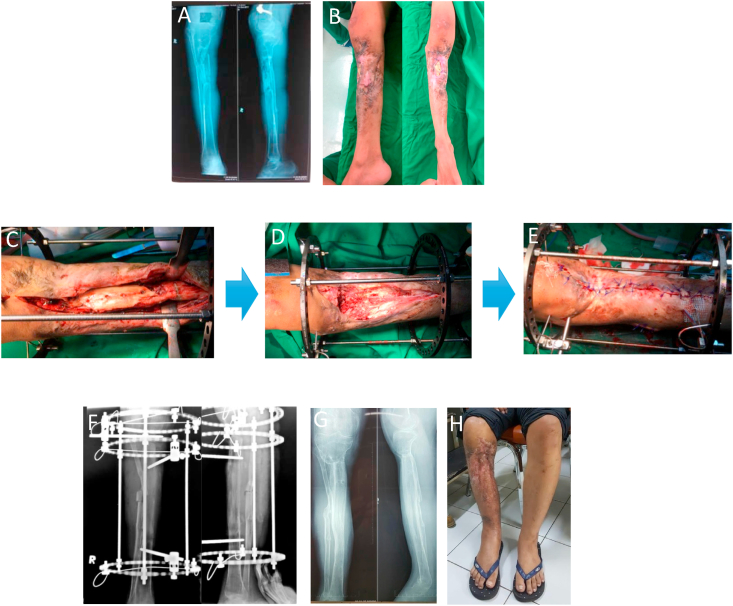
Case Illustration of a patient with chronic dead and infected bone segment and have been performed medial gastrocnemius flap (a) initial radiology (b) initial clinical picture with dead bone exposed (c) intraoperative (d) post radical resection of dead bone resulting in large bone defect and Illizarov application (e) soft tissue was closed primarily after previous undermining subcutaneously (f) docking and compression of bone transport segment upward (g) Post Illizarov removal radiograph (h) Post Illizarov removal clinical picture.

**Fig. 3:**
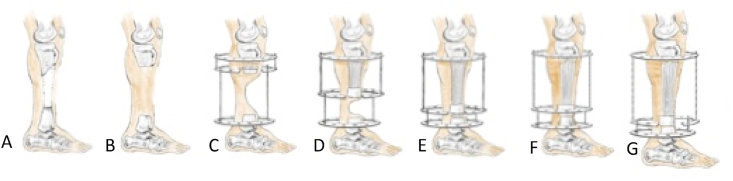
Steps of the procedure: (a) before the procedure, (b) radical debridement was performed, (c, d) bone transport procedure, (e) final result of bone transport procedure, (f) docking and soft tissue excision, and (g) bone lengthening procedure.

Illizarov method was performed to all subjects. All patients had good result with bone transport, soft tissue covered and no recurrence of infection. Three subjects had Leg Length Discrepancy (LLD) of 1 cm, whereas the others had zero discrepancy. We did not perform any bone grafting at docking site. All subjects obtained union as well as consolidation of distraction callus (See Fig. 1, Fig. 2, Fig. 3). Mean docking period of bone transport was 4.96 ± 1.69 months. No significant pain was observed, and 4 patients had ankle stiffness. Mean Foot and Ankle Disability Index (FADI) score was 84.85 ± 8.59. Mean International Knee Documentation Committee (IKDC) score was 88.36 ± 7.29 (See Table 2).

**Table 2:** Outcome.

Name	Union/Consolidation (months)	LLD	Functional Ankle Disability Index (FADI) Score (%)	International Knee Documentation Committee (IKDC) Score (%)
**ARP**	**9**	**0**	**85**	**96.6**
**AS**	**9.5**	**0**	**70**	**93.1**
**EY**	**9**	**0**	**86**	**88.5**
**MA**	**8**	**0**	**95**	**95.4**
**ME**	**8.5**	**0**	**90**	**92**
**MY**	**9**	**0**	**95**	**94.3**
**M**	**12**	**1**	**75**	**77**
**BR**	**10.5**	**0**	**90**	**89.7**
**MA**	**11**	**1**	**85**	**87.4**
**MS**	**10**	**0**	**76**	**80.5**
**GB**	**9.5**	**0**	**90**	**90.8**
**F**	**11**	**0**	**71**	**71.3**
**NA**	**10**	**1**	**86**	**88.5**
**AG**	**10.5**	**0**	**94**	**92**
	**9.82** ± **1.10**	**0.21** ± **0.42**	**84.85** ± **8.59**	**88.36** ± **7.29**

Data are presented as ^1^mean ± standard deviation if the distribution is normal and presented as ^2^median (minimum to maximum) if the distribution is not normal. Normality test was performed using Kolmogorov-Smirnov test.

## 4 Discussion

Open fracture of tibia grade 3 usually result in some following problems, such as infection, non-union, dead bone, soft tissue loss, deformities, and limb length inequalities [24]. Many of our patients had undergone several procedures (debridement, open reduction and external fixation, bone grafting, or bone spacer) and soft tissue procedure, than presented as non-union tibial fracture with infected or exposed dead bone, further implying the severity of the soft tissue defect among the patient. Adequate debridement and radical resection of dead bone segment, created long defect of bone and soft tissue. The Illizarov method is effective providing stability while enhancing soft tissue closure and filling of bony defects by bone transport procedure simultaneously [21].

In cases when flap procedure was not feasible in severe soft tissue defect, history of failed previous flaps operation, or lack of facility, radical resection of infected or dead bone segment eradicated long standing infection and created potential empty space to facilitate primary closure of soft tissue by subcutaneous undermining or rotational flap. All of our patient's wound can be closed primarily after radical debridement. Illizarov method was effective in securing infection-free union in the most difficult cases, with long defects and poor soft tissues [5]. Wound dehiscence developed in some cases, treated secondarily and healed with formation of granulation tissue followed by skin growth subsequently. In a systematic review of 24 studies, Yin et al. showed that most studies involving infected tibia non-union, the poor rate bone result was 7 % (95%CI, 0.02–0.11; I2 = 40.8 %, P = 0.119) and infectious recurrence was 6 %. The patients with infected nonunion of tibia and femur treated by Illizarov methods had a low rate of poor bone and functional results [20].

The mean of the defect gap was 14.07 ± 4.37 (6–15) cm, correlated with the docking time. The longer the gap, the more time required for docking time. We concluded that the versatility of Illizarov method can correct long bone defect and proceed with lengthening for the limb length discrepancy although it required the time and compliance from the patient.

Distraction osteogenesis through bone transport procedure performed downward, upward or both, 1 mm per day divided into four times [11]. Transport segment could creep across the defect area even though there was a narrow space or filled with granulation tissue. Near the end of transport, the skin became infold into docking site, we then surgically intervene to reconstruct the infolded skin and facilitated docking process followed by compression of bone segment. In cases where there was LLD, continued by bone lengthening procedure of the docking segment. The LLD was varied from 0 to 4 cm because muscle contraction that caused by the soft tissue defect and infection. It can interfere the bone length. The physical examination revealed large soft tissue defect that make enough gap between proximal and distal part of infected bone. The radiological examination resulted lucent between the proximal and distal end of the bone and thin soft tissue coverage covering the bone. The precise measurement of LLD to the patients was difficult because of the applied ilizarov frame. The last LLD result with the discrepancy between 0 and 1 cm was tolerable and clinically insignificant to the patient with the excellent functional outcome in the knee and ankle based on IKDC and FADI score.

Bone grafting was not performed at this procedure. Nevertheless, the union was achieved in all our patients. Aktagulu et al. reported 242 cases from 27 articles had bone union without any problems at docking site with the external fixator, without any bone grafting [31]. That further proving that Illizarov method is a very cost-effective and powerful method, in enhancing union of docking site and consolidation of distraction callus subsequently without the need of any bone graft. The advantage of this procedure in this study was not needed tertiary soft tissue procedure and additional bone graft to overcome this bone and soft tissue defect. This procedure can be done with lower medical expense for the treatment of such this condition. The other advantage of ilizarov method is the long defect of the bone can be filled with bone transport procedure without any graft added into the bone defect, including in the docking site. This can occur because of the ilizarov compression. The skin defect can also keep pace with this bone transport by primary closure. The primary closure of the skin defect is caused by approximation of the wound edge that caused by empty space after radical resection of long dead or infected bone segment without tertiary procedure. However, the disadvantages of the ilizarov technique is pin track infection and failure in distraction although the occurrence of these incidence is relatively minimal.

Throughout the procedure, there was no recurrent of infection, nor significant pain. Nearly all patients had excellent FADI score and IKDC evaluation. Four patients had ankle stiffness due to very severe injury with lower FADI score (range from 70 to 76 points), long standing immobilization and inadequate ankle exercise.

## 5 Conclusion

Open fracture of tibia grade 3 often results in very difficult-to-solve complications. Several operative procedures were previously undergone, but ended up with non-union and exposed long dead bone or infected segment. The Illizarov method can effectively address long bone and soft tissue defect since radical debridement created potential empty space and infected free area that allowed soft tissue closure primarily or secondarily without the need for tertiary soft procedure, while bone transport gradually filled bone defect subsequently, then later continued with bone lengthening to correct limb length discrepancy. Compression at docking site enhanced union without the need of bone graft, as well as distraction callus consolidation.

## Conflict of interest

The authors have no conflict of interest to disclose.

## Funding

This research did not receive any specific grant from funding agencies in the public, commercial, or not-for-profit sectors.

## Ethical approval

Ethical approval was not required in the treatment of the patient in this report.

## Informed consent

Written informed consent was obtained voluntarily from the parents for publication of this case and accompanying images. A copy of the written consent is available for review by the Editor-in-Chief of this journal on request.

## Declaration of competing interest

The authors stated that there is no conflict of interest to disclose.

## Registration of research studies

1. Name of the registry: UMIN
2. Unique Identifying number or registration ID: R000050872
3. Hyperlink to your specific registration (must be publicly accessible and will be checked): UMIN000044523
